# Early warning and response system (EWARS) for dengue outbreaks: Recent advancements towards widespread applications in critical settings

**DOI:** 10.1371/journal.pone.0196811

**Published:** 2018-05-04

**Authors:** Laith Hussain-Alkhateeb, Axel Kroeger, Piero Olliaro, Joacim Rocklöv, Maquins Odhiambo Sewe, Gustavo Tejeda, David Benitez, Balvinder Gill, S. Lokman Hakim, Roberta Gomes Carvalho, Leigh Bowman, Max Petzold

**Affiliations:** 1 Occupational and Environmental Medicine, Sahlgrenska Academy, University of Gothenburg, Gothenburg Sweden; 2 Special Programme for Research and Training in Tropical Diseases (TDR), World Health Organization, Geneva, Switzerland; 3 Centre for Medicine and Society/ Anthropology, Uniklinikum Freiburg, Freiburg, Germany; 4 Department of Public Health and Clinical Medicine, Epidemiology and Global Health, Umeå University, Sweden; 5 Institute of Public Health, Heidelberg University, Heidelberg, Germany; 6 Ministry of Health, Mexico City, Mexico; 7 Information and Documentation Surveillance section, Disease Control Division, Ministry of Health, Putrajaya, Malaysia; 8 Office of the Deputy Director General of Health (Public Health), Ministry of Health, Level 12, Block E7, Federal Government Administrative Complex, Putrajaya, Malaysia; 9 School of Medicine, International Medical University, Bukit Jalili, Kuala Lumpur, Malaysia; 10 Secretaria de Vigilância em Saúde, Ministério da Saúde, Brasília, DF, Brasil; 11 Umeå Centre for Global Health Research, Umeå University, Sweden; 12 Health Metrics Unit, Sahlgrenska Academy, University of Gothenburg, Gothenburg, Sweden; Columbia University, UNITED STATES

## Abstract

**Background:**

Dengue outbreaks are increasing in frequency over space and time, affecting people’s health and burdening resource-constrained health systems. The ability to detect early emerging outbreaks is key to mounting an effective response. The early warning and response system (EWARS) is a toolkit that provides countries with early-warning systems for efficient and cost-effective local responses. EWARS uses outbreak and alarm indicators to derive prediction models that can be used prospectively to predict a forthcoming dengue outbreak at district level.

**Methods:**

We report on the development of the EWARS tool, based on users’ recommendations into a convenient, user-friendly and reliable software aided by a user’s workbook and its field testing in 30 health districts in Brazil, Malaysia and Mexico.

**Findings:**

34 Health officers from the 30 study districts who had used the original EWARS for 7 to 10 months responded to a questionnaire with mainly open-ended questions. Qualitative content analysis showed that participants were generally satisfied with the tool but preferred open-access vs. commercial software. EWARS users also stated that the geographical unit should be the district, while access to meteorological information should be improved. These recommendations were incorporated into the second-generation EWARS-R, using the free R software, combined with recent surveillance data and resulted in higher sensitivities and positive predictive values of alarm signals compared to the first-generation EWARS. Currently the use of satellite data for meteorological information is being tested and a dashboard is being developed to increase user-friendliness of the tool. The inclusion of other *Aedes* borne viral diseases is under discussion.

**Conclusion:**

EWARS is a pragmatic and useful tool for detecting imminent dengue outbreaks to trigger early response activities.

## Introduction

Dengue is currently the fastest-spreading mosquito-borne viral illness. It has become a leading cause of morbidity in children and adults in many tropical and sub-tropical countries [1]. Disease outbreaks (epidemics) overburden stretched health systems, impoverished societies and households alike.

The transmission of dengue and other *Aedes*-borne arboviral diseases can only be controlled through effective vector control interventions, potentially combined with an efficacious vaccine [2]. However, the required level of interventions to prevent transmission has not been achieved and outbreaks have become increasingly frequent in the past two decades, more recently also joined by other *Aedes* borne diseases such as chikungunya and Zika [3, 4]. Epidemics are usually detected too late when the case numbers are already growing unrestrained. It is argued that early outbreak warning and response may be crucial for mitigating or averting the destructive consequences of outbreaks [5].

For other communicable diseases of epidemic potential, research on outbreak warning has often been restricted to the definition of an epidemic as compared to endemic transmission (Rift Valley Fever [6], malaria [7], influenza [8]), or limited to alarms under extreme meteorological conditions [9] such as ENSO (El Nino Southern Oscillation). ENSO is a coupled ocean-atmosphere mode of variability, which can produce extreme climate conditions such as heavy rainfall, which can boost the breeding places for disease vectors such as *Aedes* mosquitoes. [10]. However, early outbreak warning using alarm indicator(s) to trigger early response has rarely been studied [5].

In order to address the need, for an alarm system for dengue outbreaks, the Special Programme for Tropical Disease Research and Training (WHO/TDR) initiated together with national dengue control services and academia in partner countries the development of an early dengue outbreak warning and response system (EWARS), jointly supported by the EU-funded IDAMS consortium. The stepwise development of the EWARS method and its retrospective testing with preliminary results have been previously published [11].

The EWARS model is based on the Shewhart method, which is typically applied in industrial and business settings [12], but more increasingly in the context of public health [13]. When applied to dengue, the EWARS adopts systematic control charts, using the historic mean and standard deviation (SD) of the dengue outbreak. This defines the Endemic Channel which represents the number of dengue cases within the expected normal range or the ‘in-control’ state, while anything above this moving average is considered representative of an unusual number of cases and, an ‘out-of-control’ state (i.e., an outbreak). Although the use of the Endemic Channel applying a fixed 2 SD threshold for disease surveillance has been reported in some countries [14], the EWARS tool includes locally valid alarm signals and thresholds both for alarm indicators and dengue cases [11]. This early warning model does not merely rely on outbreak records but utilizes a broad spectrum of epidemiological, entomological and meteorological data to inform about a forthcoming dengue outbreak. The EWARS tool has more recently been complemented by a computer-assisted user’s work book (WHO 2017) [15].

In the context of EWARS incorporation into routine national surveillance systems, there was an opportunity to advance both the technical and operational components via feedback from participating countries that ensures the tool becomes increasingly user-friendly.

In this paper, we summarize these recent adaptation efforts guided by the experience of local health managers when applying the tool and by further statistical analyses that underpin the recent modifications.

## Methods

### Study settings and data collection

#### Qualitative assessment of field experience of the first generation of EWARS

The first generation of the EWARS tool identified evidence-based meteorological, epidemiological and entomological indicators predicting a forthcoming outbreak [16]. These indicators were later tested across ten districts in each of the three participatory countries in the prospective study: Brazil, Malaysia and Mexico. The information on the plausibility of applying the tool in routine surveillance, user-friendliness and “lessons learned” were collected through a self-applied questionnaire following a seven to ten-months testing period, from December 2016 to February 2017. This was complemented by data from personal semi-structured interviews. Participants in these interviews were staff members from the vector control, epidemiology and health promotion units covering all localities/ districts where the EWARS tool is being implemented. Among the participants are biologists, physicians and social communicators. In Mexico, all staff from different components of dengue surveillance and response were invited to voluntarily and anonymously conduct the semi-structured interviews through organized meetings. In Brazil and Malaysia, however, all recruited staffs were sent these semi-structure interviews via email. These interviews included 17 open-ended questions covering the different components of the EWARS tool and health officers’ opinions/ satisfaction regarding its prospective application. Qualitative content analysis was used as methodology. Concepts were obtained from the interviews and systematically compared with the rest of interviews using a technique from the Grounded Theory, i.e. the constant comparisons technique [17]. These concepts were compared and grouped throughout the interviews accounting for patterns and variations. The references selected are representative of the most frequently shared concepts that arose from the interviews. Participation was voluntary and confidentiality was assured to the participants; no personally identifiable information was recorded.

### Developing the second generation of the EWARS tool

**Fig 1:**
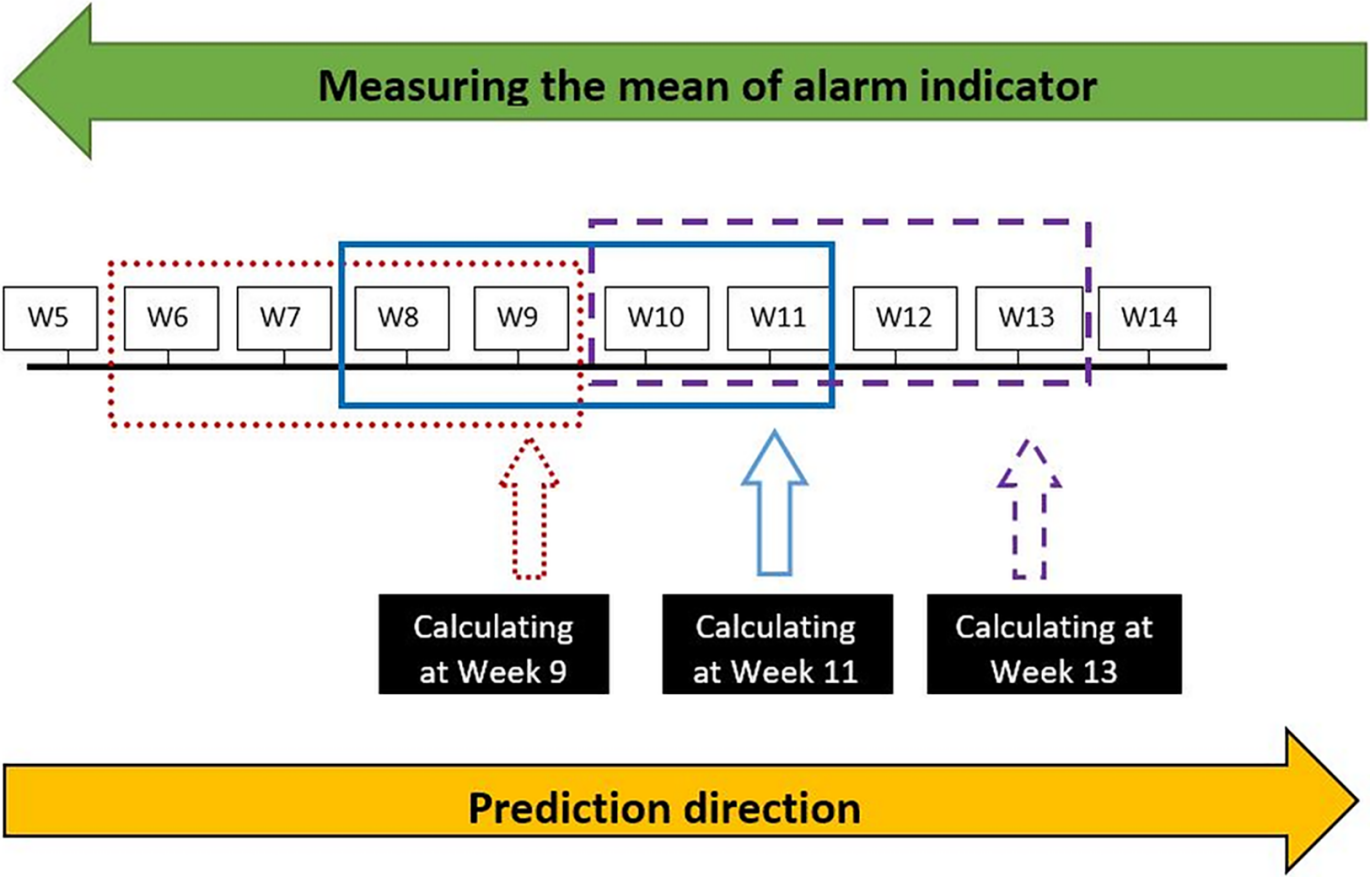
The mechanism of calculating the mean of alarms indicators. The mean of each alarm indicator was consecutively measured from the point of estimate (W = epidemiological week) and for a preceding number of weeks. The window size of the alarm indicator is objectively set to define the appropriate length of the preceding period when calculating the mean alarm indicator (e.g. choosing alarm window size of 4, this step will measure the mean of each alarm indicator during the last four consecutive weeks including the week we are measuring from). The calculated alarm mean then enters the logistic regression model as a predictor of an outbreak.

**Fig 2:**
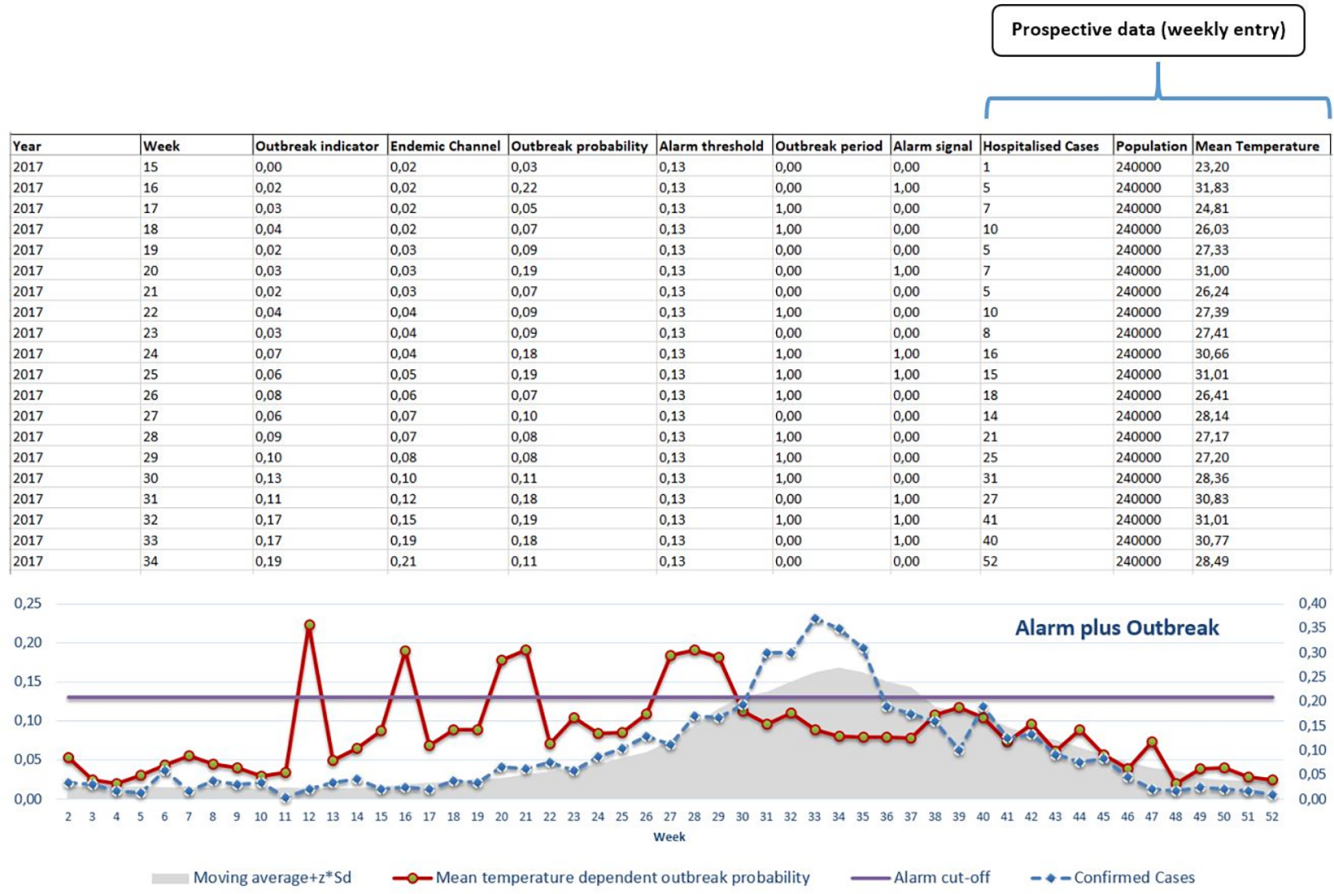
Prospective data entry and interpretations based on coefficients and outbreak probability from retrospective data. Columns with indications by blue arrows are locations for prospective data entry. Alarm signal column (“0” is no alarm signal and, “1” is alarm signal) would inform about a forthcoming outbreak.

**Fig 3:**
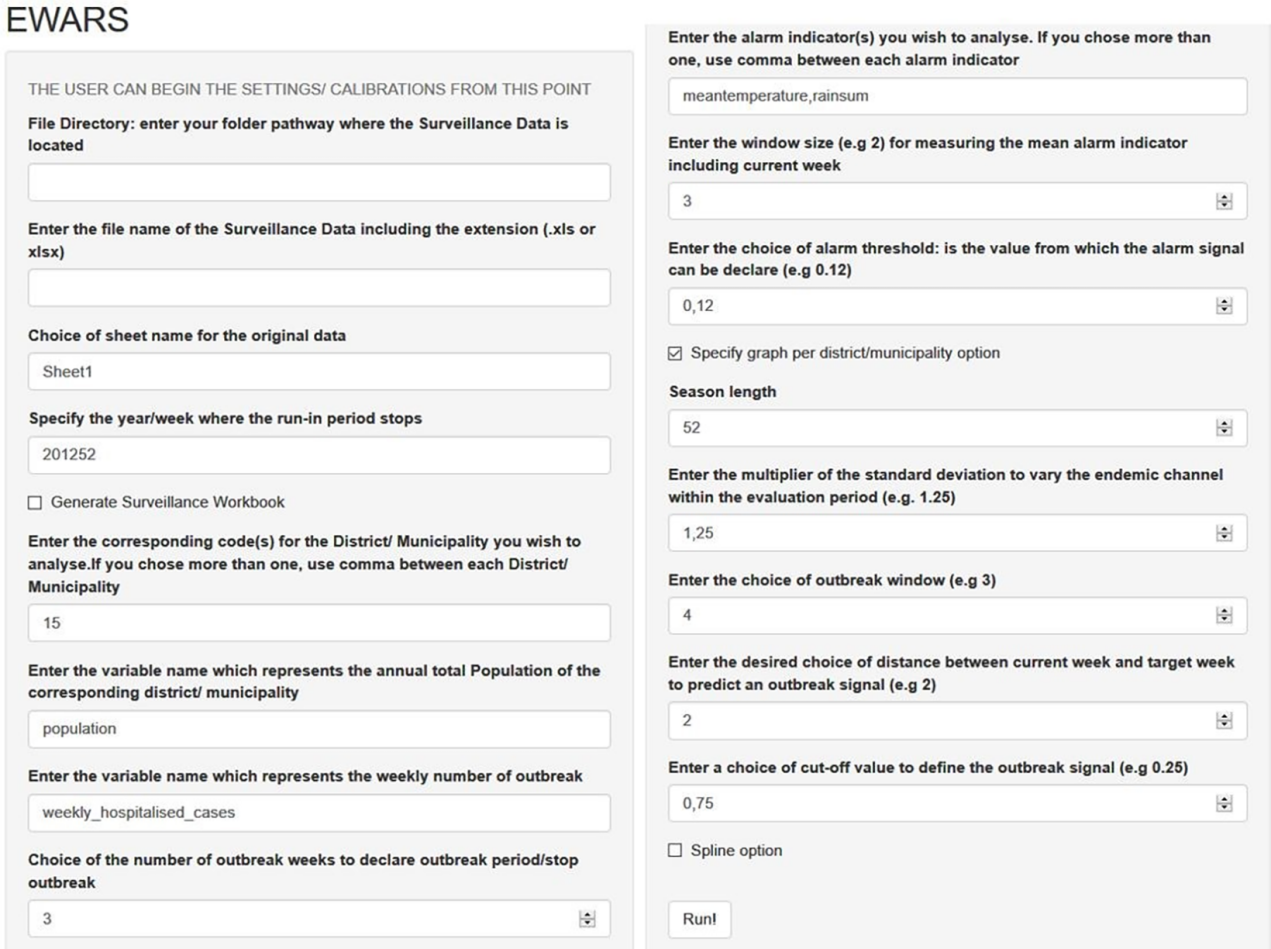
The user’s interface in the second generation EWARS-R.

Based on recommendations from users’ of the first-generation testing, the following adaptations were introduced into the advanced EWARS-R tool:

- Change from STATA software (StataCorp, 2015. Release 14.1, which is not an open-source and consequently limits its accessibility) to “EWARS-R”, using the open-access “R” software (which is free and equally compatible with common programming languages).
- District-specific alarm data analysis rather than use of overall country information,
- More accurate surveillance information: The run-in data (the first half of the retrospective surveillance data, used to develop and calibrate the prediction model) utilized broader range of weekly records and was defined by all records preceding the year 2009 in Brazil, 2012 in Malaysia and 2014 in Mexico. All data records reported after these cut-off points (years) were used as the evaluation data (i.e. the second half of the retrospective surveillance data, used to validate the derived parameters from the prediction model, and to identify the correct and false outbreak alarms), which facilitates a multiple rounds of cross-validation process.
- Reduced duration of the period of the “moving average” (from 13 to 7 weeks): Average (mean) number of dengue cases–within the expected “normal” or seasonal range–were calculated for a fixed window size of three preceding and three succeeding weeks from the point of measure 3+1+3 weeks to generate a smoothed “moving average”. Relatively smaller window size would likely reveal more details about the outbreak indicator.
- Extended number of candidate alarm indicators (meteorological, entomological and epidemiological) which now includes: i) Ovitrap-index in Mexico (the proportion of positive ovitraps and the mean number of eggs per block of houses- the only available entomological indicator with weekly reporting) and, ii) Predominant dengue virus serotype in Malaysia (indicating changes of serotype; weekly information on this indicator was previously unavailable in any of the study countries). Fig 1 illustrates the mechanism of calculating the mean of alarms indicators before entering the regression model.
- Advanced method of analysis. The second generation EWARS-R has advanced into multiple logistic regression modelling of outbreak probability, allowing one or more alarm indicator(s) per analysis. This has the potential to account for missing or inconsistent records and improve the explanation of the outbreak prediction. Another analytical advancement with potential for model enhancement is the inclusion of the ‘Spline’ function to adjust for likely non-monotonic nature in some variables. A non-monotonic relationship occurs when the number of dengue cases take an inversed direction as the mean alarm indicator continue to increase, during a particular period. For instance, the number of dengue cases increase as the environmental temperature increases but at one point, a decrease in dengue cases is observed due to a decreased vector activity as a result of excessive temperatures.
- Use of district-level Excel spreadsheet files (prospective analysis) containing the algorithm and all parametric coefficients needed for calculating the outbreak probability: These coefficients are estimated during the retrospective phase, depending primarily on the sensitivity (i.e. the proportion of correctly predicting an outbreak out of all outbreaks) and PPV (i.e. the proportion of correct alarms out of all alarms) as direct measures for deciding the best calibrated settings (i.e. those with highest sensitivity and PPV). In the Excel spreadsheet file, the prospective information of alarm indicators is being entered to estimate the probability of an ‘alarm signal’ for a forthcoming outbreak—an alarm signal is triggered when the outbreak probability crosses a given alarm threshold. This process is achieved by inserting weekly district-level prospective data of outbreak cases and alarm indicators (see example in Fig 2). In these excel spreadsheets, displayed parameters (top-left hand corner) and the graph can guide the interpretation of the “alarm signal” field. For instance, as illustrated in Fig 2, prospective information on “mean temperature” in week 12 shows an “alarm signal” predicting a forthcoming outbreak in the following 2 weeks (prediction distance, lag time) from the current week.
- Implementation of an external interface to reduce human error and increase users’ acceptability. The shift from STATA into EWARS-R facilitated the migration from the typical programming-based STATA do-file interface [15] to a more user-friendly interface designed to focus user’s data inputs to specific ‘boxes’ of interest during the process of the program setting and data calibration, see Fig 3.

### Quantitative assessment of the second generation of EWARS-R

Brazil, Malaysia and Mexico were once again selected as study countries providing convenient contextual, environmental as well as operational information to test the newly modified EWARS-R tool. Surveillance data were collected on relevant outbreak and alarm indicators covering data records for the period 2007–2016 in Brazil, 2003–2016 in Malaysia and 2009–2016 in Mexico. Data collection, which was based on the country’s routine surveillance system, has more recently [5, 18] been integrated by a more standardized protocol to ensure compliance and high-quality data reporting. Nonetheless, dataset quality varied across the three participating countries, mostly due to different contextual and operational practices, resulting in some inconsistent or undefined records being excluded prior the analysis.

As meteorological stations were not available in all health districts, Mexico and Brazil organized an improved access to meteorological information and additionally the Mexican Ministry of Health (MoH) provided meteorological stations to the participating district health offices. In parallel efforts in Malaysia, external websites were additionally included to data sets from local meteorological stations [19, 20]. The “epidemiological week” (Sunday to Saturday) was set as the temporal unit (annual) for data mapping after excluding weeks 1 and 53 from all datasets due to inconsistent data quality whereas, districts/ municipality were defined as the spatial units. Based on these temporal and spatial units, data collection process used the Excel spreadsheet as a convenient format to consistently store the following sets of variables:

- **Meteorological variables**: outdoor mean air temperature, outdoor mean humidity and total rainfall;
- **Epidemiological variables**: mean age of hospitalized cases, serotypes (available from Malaysia only due to late reporting in the other countries), probable, and laboratory confirmed and hospitalized dengue cases. Dengue cases (hospitalized cases used as outbreak indicator) were measured per 1000 population (annual) of the corresponding spatial units;
- **Entomological variables**: Ovitrap Index (Mexico only [21]), measured as both the proportion of positive ovitraps and mean number of ovitraps per block (i.e. infested with *Aedes* larvae/pupae). (Note: neither the Breteau Index (BI) nor the House Index (HI) could be used due to inconsistent data and reporting schedules.

Based on recommendations from previous EWARS report [11], we used a range of 1.0–1.5 of ‘z’ value to define our Endemic Channel and thus, an ‘outbreak week’ (i.e. state of out-of-control) was triggered once dengue incidence crossed the upper threshold of the Endemic Channel (z*SD). Although there is not much evidence on mean lag times, based on findings from other reports [22, 23] and further consultations with expert panels, a range of 1–12 weeks for outdoor air temperature, 3–12 weeks for rainfall, 2–12 weeks for outdoor humidity and 2–8 weeks for Ovitrap Index and serotype were used in the prediction process as lag-times from the state of alarm to the beginning of the outbreak. Single and multiple logistic regression using a list of alarm indicators were performed to estimate the ‘outbreak probability’ needed to define the ‘alarm signal’ at district level. Using the recommended range of 0.08–0.2 for alarm threshold [11], an ‘alarm signal’ was triggered when the derived outbreak probability crossed the ‘alarm threshold’. For this quantitative assessment of the second generation EWARS-R, we sought simple descriptive statistics to present average sensitivity and PPVs separately for each country. The Free R software version 3.4.3 was used in this analysis.

### Ethical approval

Ethical approval for the study protocol was granted by WHO Regional Ethical Committees, specifically the Pan American Health Organization Ethics Review Committee (PAHO-ERC; Ref No. 2011-12-0021) and the Western Pacific Regional Office Ethics Review Committee (WPRO-ERC; Ref. 2013.25.ICP.2.ESR). These approvals were accepted by national MoHs.

## Results

### User assessment of the first generation EWARS

A total of 34 district health managers responded to the survey and answered questions during these semi-structured interviews in Brazil (n = 7), Malaysia (n = 10) and Mexico (n = 17). Table 1 summarizes all responses. There was an overall consensus towards the usefulness of the EWARS tool in averting or mitigating dengue outbreaks and its potential use with other *Aedes*-borne diseases. Health managers equally acknowledged the benefits of EWARS training courses and the technical support provided by tutors. User opinions about the mode of data collections and interpretations were positive and respondents viewed it as appropriate for their corresponding settings. Some operational aspects concerning data availability and the post-outbreak responsiveness, however, remain to be fully addressed. According to the interviewees, the mechanism of case notification needs to be enhanced and obtaining timely meteorological data is yet a challenge. Several recommendations have been taken on board for updating the tool, particularly the use of smaller geographical units, the use of entomological indicators (ovitrap data from Mexico as these was the only systematically collected entomological information with timely reporting) and the combination of indicators (allowing for multiple environmental, entomological and epidemiological indicators to predict an outbreak probability).

**Table 1:** EWARS after 7 to 10 months of use in Brazil (7 district officers), Malaysia (10 district officers) and Mexico (17 district officers).

EWARS component	General opinion	Room for improvement	Representative quotes
Does the EWARS tool reduce dengue outbreaks?	**Brazil:** All participants think it is very useful to reduce outbreaks. **Malaysia:** There is a high agreement that the EWARS tool is useful for reducing dengue outbreaks **Mexico:** The majority of the interviewees agree that EWARS is really helpful to anticipate outbreaks and reduce the dengue burden.	**Brazil:** There are many external circumstances that are affecting the tool’s prediction (lack of resources, data opportunity, and intersectoral work). **Malaysia:** The spatial dimension should be smaller than districts. **Mexico:** Resources are needed to carry out all the activities.	**Brazil:** “It has the potential, if done properly it should serve. It is important to recognize the importance of intersectoral work […]. It is not a problem of the tool, but it is a problem for the operation of the tool in a reality that is difficult to change” (participant brz352). **Malaysia:** “Yes, early detection and response tool helps, but it should be done according to localities in the district itself” (participant mal397) **Mexico:** “It truly is helpful, it makes us realize what to do when an outbreak is coming” (participant esp2112)
Other benefits	**Brazil:** This tool can be used for other arboviruses; it has a motivational effect for the vector control staff. **Malaysia:** EWARS tool can be used for risk communication to other stakeholders. **Mexico:** It could also benefit the fight against other arboviruses and it also strengthens the institution as it forces all the components to work together and pay continuous attention to the indicators.	Explore options for extending the tool to other Aedes-borne diseases	**Brazil:** “The study was a challenge, forced people out of the comfort zone and moved all the work” (participant brz986). **Malaysia:** “You can explain to other parties concerned about an outbreak Scientifically/ graphically, especially communities or local councils / local leaders” (participant mal288). **Mexico:** “Yes, it can be also helpful to alert over other diseases such as Chikungunya and Zika” (participant esp759)
Strengths	**Brazil:** It anticipates a dengue outbreak indicating what response is adequate, and thus reducing the cost involved **Malaysia:** Dengue outbreaks can be detected in advance allowing a timely and more efficient & effective response **Mexico:** 1) It alerts with enough time to take anticipatory actions and therefore to reduce the outbreak burden. 2) It strengthens team work and intersectoral cooperation 3) It has the potential to be used for other arboviruses.	Improve strengths by improving the country surveillance system and by further developing the EWARS tool	**Brazil:** “Strengths are: timely detection, time to organize a response, and cost savings” (participant brz187) **Malaysia:** “Able to warn the upcoming outbreak” (participant mal003); “…can detect increments of cases by changes of temp” (participant mal397). **Mexico:** “The main strength is the great accuracy in the outbreak prediction, we observed it was of 6 weeks” (participant esp8741)
Weaknesses	**Brazil:** 1) Delay of incoming data 2) Lack of resources. 3) Lack of intersectoral work. 4) It does not take into account the peculiarities of each locality. **Malaysia:** 1) Delay in obtaining the meteorological data. 2) The spatial scale of the study (district) should be smaller. 3) Current Dengue outbreak definition (“2 cases in a locality within 2 weeks”) does not apply 4) It can give false alarms. **Mexico:**1) Lack of data timeliness. 2) Lack of resources to carry out the staged response. 3) Lack of engagement of other sectors.	Improve calculations of PPV and sensitivity	**Brazil:** “It works with probable cases: the notification network has to be very well structured. Because if there is no notification of cases, you will not have time to do the analysis as an alert system” (participant brz662) **Malaysia:** “Cannot detect increase of cases within localities in districts using EWARS; Late met data” (participant mal288). **Mexico:** “The time of sending the information is maybe a bit late because of lack of time or resources” (participant esp172)
Indicators	**Brazil:** The indicators used in the study are appropriate. **Malaysia**: The majority of the interviewees think that mean age is not appropriate, but positive opinions were related to indicators such as temperature and, in a lesser proportion, humidity. **Mexico:** The indicators selected are adequate for the objectives of the study.	**Brazil:** Temperature is the weakest indicator, it would be good to include entomological data & combined indicators. **Malaysia:** Dengue definitions should be revised (DF/DHF/SS classification still in use). **Mexico:** Include other indicators & combinations of indicators.	**Brazil:**” Yes, they are appropriate, […] I think we should also use entomological indicators” (participant brz662) **Malaysia:** “Only mean temperature and humidity are appropriate indicators that can be used” (participant mal643) **Mexico:** “The indicators used are the right ones because they help to intervene timely before a possible outbreak” (participant esp125)
Data collection sheet	**Brazil:** Sheet is appropriate **Malaysia:** Almost all participant agree that the data collection sheet is appropriate **Mexico:** Participants answered almost unanimously that it is appropriate.	**Brazil:** Temperature is difficult to obtain. Lack of timely epidemiological data. **Malaysia:** Difficulties to obtain meteorological data. **Mexico:** Eliminate the indicators not used.	**Brazil:** “I would not change the data collecting sheet. The bad thing is the delay in collecting and consolidating daily and weekly temperatures” (participant brz459) **Malaysia:** “Yes [it is appropriate]. You can identify and compare data submitted” (participant mal8675). **Mexico:** “I had no troubles, it is very easy to fill in” (participant esp172)
Diagram for prospective monitoring	**Brazil:** They are fine and participants would not change anything at all. **Malaysia:** All participants see the graphs as a proper way to analyze the data. **Mexico:** All participants are satisfied with the current way of presenting the information.	**Brazil:** To include additional signals when an alarm is above threshold. **Malaysia:** It would be better to have graphs by locality instead of district level. **Mexico:** No room for improvement was mentioned	**Brazil:** “They are easy to understand, it is fine” (participant brz986). **Malaysia:** “Yes, the graphs were easy to use because it was already automatic plotted when the data was typed in the excel format” (participant mal003) **Mexico:** “…they are very clear and show us everything to detect an outbreak timely, I would not change anything” (participant esp2112).
Staged response	**Brazil:** Most people agree with the staged response. **Malaysia:** Most participants see the staged response to be adequate. **Mexico:** The staged response is adequate.	**Brazil:** It should be more aligned with national guidelines. **Malaysia:** The staged response should be aligned with the current routine response. **Mexico:** To promote more intersectional work.	**Brazil:** “Inconsistent with the national and local contingency plan. The language of this is better than that of the ministry (level 0, 1, 2, 3).” (participant brz746) **Malaysia:** “Yes, the graphs were easy to use because it was already automatic plotted when the data was typed in the excel format” (participant mal003) **Mexico:** “It should be the only response implemented in all the department” (participant esp759)
Response sheet	**Brazil:** It is difficult to understand and to fill in. **Malaysia:** Almost all districts approved it. **Mexico:** It is a bit confusing as it is difficult to distinguish between routine activities and early response activities.	**Brazil:** Avoid duplication of work as there is another similar worksheet that is requested by the government. **Malaysia:** It is not clear when the control actions are finished **Mexico:** Adding more options, more activities	**Brazil:** “I found this worksheet very difficult to fill in. It generates doubts and facilitates wrong completion” (participant brz662) **Malaysia:** “Appropriated. No change required.” (participant mal882) **Mexico:** “It is good, but adding more response options would be ideal to specify the actions” (participant esp125).
Training course and technical assistance	**Brazil:** Everybody received training course at the beginning but there was little support afterwards. **Malaysia:** A training course was carried out at national level. **Mexico:** The training course was adequate and good technical support by email or telephone.	**Brazil:** Translation during the training course and proper follow up to emerging doubts. **Malaysia:** There is the need of replication at district level. **Mexico:** To spend more time in the training.	**Brazil:** “There are some doubts, when you start implementing the doubts arise. It would have been better to have more support for clarifying the doubts” (participant brz746). **Malaysia:** “It is adequate for persons who are in charge i.e. MOH and Surveillance Officer, but no training was given to ground staffs who are doing the control activities” (participant mal913). **Mexico:** “It was good, but it would be helpful to dedicate more time to explain how to fill in the response questionnaire…” (Participant esp759).

### Sensitivities and PPVs in the second generation EWARS version (using more recent data sets)

Compared to the first generation of EWARS, the updated version demonstrated in all three countries an increased sensitivity and PPVs (Tables 2–4). The sensitivity of correctly predicting an outbreak ranged between 83–99% in Brazil, 50–99% in Malaysia and 79–100% in Mexico. PPVs ranged between 40–88% in Brazil, 71–80% in Malaysia and 50–83% in Mexico. The analysis of serotype (using two-week lag-time) and ovitrap variables (using three-week lag-time) showed promising findings with sensitivity and PPVs of 50% and 71% for serotype and 79% and 60% for ovitrap, respectively.

**Table 2:** Sensitivity and PPV for outbreak detection using hospitalized dengue cases as outbreak indicator in relation to earlier weekly changes in alarm indicators in Brazil.

Alarm Indicator	Sensitivity (%)	Positive Predictive Value (%)
Mean temperature (Celsius)	91	65
Total rainfall (cm)	88	88
Mean age (years)	92	84
Probable cases	99	59
Humidity	89	71
Multiple indicators*	83	40

*Temperature, rainfall, mean age, probable cases & humidity were used altogether in a multiple analysis

**Table 3:** Sensitivity and PPV for outbreak detection using hospitalized dengue cases as outbreak indicator in relation to earlier changes in alarm indicators in Malaysia.

Alarm Indicator	Sensitivity (%)	Positive Predictive Value (%)
Mean temperature (Celsius)	99	80
Mean age (years)	97	75
Change of predominant serotype	50	71
Multiple indicators*	99	80

*Temperature, mean age & serotype were used altogether in a multiple analysis

**Table 4:** Sensitivity and PPV for outbreak detection using hospitalized dengue cases as outbreak indicator in relation to earlier changes in alarm indicators in Mexico.

Alarm Indicator	Sensitivity (%)	Positive Predictive Value (%)
Mean temp (Celsius)	81	72
Total rainfall (cm)	87	65
Mean age (years)	89	74
Probable cases	100	83
Ovitrap (%positive traps)	79	60
Ovitrap (avg. no. of eggs per block)	76	50
Humidity	94	50
Multiple indicators*	84	77

*Temperature, rainfall, mean age, probable cases, positive ovitrap & humidity were all used in the multiple analysis

During the calibration and validation process, the new tool proved better in handling inconsistent data, for instance when data records in one variable are reported as both absolute values and fractions. Improvement of the operational aspects of the modified version also targeted situations when data records on outbreak indicator (hospitalized cases) in some spatial units were merely “zero”, which would usually cause systematic error when running the binary regression. Moreover, the processing rate of the new tool is around 30 second per run using an ordinary computer, which is almost 10 times faster than the older version–an essential element for this process. The user-friendly interface and instant “error/ solution” messages show potentials for increased acceptability of applying such tool in different settings. Indeed, a significant trend towards improved data quality and reporting has been documented during this study.

## Discussion

### Achievement of updating the EWARS tool

When developing the first version of the EWARS tool [11], the ability of an extended list of candidate alarm indicators [5] for outbreak prediction was assessed but had to be reduced to a few viable indicators. The subsequent development of the EWARS-R tool was progressing towards model enhancement by processing multiple meteorological, epidemiological and entomological indicators but was also limited by the lack of robust local information on a weekly basis. One of the persisting challenges is access to real-time meteorological data. The use of satellite data for surface temperature and rainfall as well as a more formalized collaboration between MoH and meteorological institutions has been initiated. Furthermore, testing the previously-mentioned installation of robust meteorological stations in Mexico is currently underway. Another important challenge is to integrate EWARS-R into national electronic surveillance systems which has the potential to avert double recording of data (one for the routine surveillance and one for EWARS practice) thus, increased efficiency and consistency.

Progression with this project provided clear indications that interested countries should allocate appropriate resources into a functioning and high-quality electronic surveillance system that can allow detection and response to outbreaks at early stages [24]. Furthermore and against the background of the Sustainable Development Goals (SDGs), which will involve major investments in health by many countries, robust indicators for tracking health systems’ effectiveness will be critical. EWARS-R is therefore, an example of a tool able to contribute directly to that process.

### Progressive developments towards a widespread use of the EWARS-R tool

The use of the open-access software package “R” have made the tool more user-friendly, with improved sensitivity and PPVs for early outbreak predictions, and its open source web-based applications encourages further developments, which are underway.

The transformation of software application (from STATA to “R”) is coupled with the development of an integrated web-based dashboard to facilitate a user-friendly interface and allow instant reporting and sharing of model coefficients for the prospective data entry. This can potentially augment the scale-up plan of this automated tool and support current calls for its widespread use mainly in critical settings [25]. More details about the development of the web-based dashboard will be presented in a separate paper including web-links for obtaining this freely-available tool.

National guidelines for a staged response (initial, early, late and emergency response to a dengue outbreak) were already incorporated in previous phases of this study [24], and carried forward into the current EWARS-R. Despite systematic integration of this action plan in the new EWARS-R, which can routinely report about appropriate response stage, further research is needed to provide evidence of the operational and cost-effectiveness of these actions and to develop clear indications about priority areas where to start and focus response actions when an alarm signal is reported (“hot spots”) [25].

## Conclusion

The EWARS system is a pragmatic solution for countries to mount during the pre-outbreak phase as an effective dengue outbreak response defined by alarm signals. The knowledge gained during the calibration and the prospective phases of development on operational, technical and statistical issues form the basis for promoting its scaling up and wider use in dengue outbreak prone countries.

## Supporting information

S1 File Questionnaire items for the qualitative assessment of field experience of first generation EWARS. (PDF)

## References

[1] Murray NEA, Quam MB, Wilder-Smith A. Epidemiology of dengue: past, present and future prospects. Clinical epidemiology. 2013;5:299 doi: 10.2147/CLEP.S34440 23990732 10.2147/CLEP.S34440 PMC3753061

[2] World Health Organization. Dengue vaccine: WHO position paper -July 2016. Geneva; 2016. Contract No.: 30. 10.1016/j.vaccine.2016.10.070 28185744

[3] Bowman LR, Donegan S, McCall PJ. Is dengue vector control deficient in effectiveness or evidence?: Systematic review and meta-analysis. PLoS neglected tropical diseases. 2016;10(3):e0004551 doi: 10.1371/journal.pntd.0004551 26986468 10.1371/journal.pntd.0004551 PMC4795802

[4] Lowe R, Stewart-Ibarra AM, Petrova D, García-Díez M, Borbor-Cordova MJ, Mejía R, et al Climate services for health: predicting the evolution of the 2016 dengue season in Machala, Ecuador. The Lancet Planetary Health. 2017;1(4):e142–e51. 10.1016/S2542-5196(17)30064-5 29851600

[5] Badurdeen S, Valladares DB, Farrar J, Gozzer E, Kroeger A, Kuswara N, et al Sharing experiences: towards an evidence based model of dengue surveillance and outbreak response in Latin America and Asia. BMC Public Health. 2013;13(1):607. 23800243 10.1186/1471-2458-13-607 PMC3697990

[6] Anyamba A, Chretien J-P, Small J, Tucker CJ, Formenty PB, Richardson JH, et al Prediction of a Rift Valley fever outbreak. Proceedings of the National Academy of Sciences. 2009;106(3):955–9. 10.1073/pnas.0806490106 PMC2626607 19144928

[7] Hay SI, Simba M, Busolo M, Noor AM, Guyatt HL, Ochola SA, et al Defining and detecting malaria epidemics in the highlands of western Kenya. Emerging infectious diseases. 2002;8(6):555 doi: 10.3201/eid0806.010310 12023909 10.3201/eid0806.010310 PMC2738480

[8] Vega T, Lozano JE, Meerhoff T, Snacken R, Mott J, Ortiz de Lejarazu R, et al Influenza surveillance in Europe: establishing epidemic thresholds by the moving epidemic method. Influenza and other respiratory viruses. 2013;7(4):546–58. doi: 10.1111/j.1750-2659.2012.00422.x 22897919 10.1111/j.1750-2659.2012.00422.x PMC5855152

[9] Reiner RC, King AA, Emch M, Yunus M, Faruque A, Pascual M. Highly localized sensitivity to climate forcing drives endemic cholera in a megacity. Proceedings of the National Academy of Sciences. 2012;109(6):2033–6. 10.1073/pnas.1108438109 PMC3277579 22308325

[10] Gagnon AS, Smoyer-Tomic KE, Bush AB. The El Nino southern oscillation and malaria epidemics in South America. International Journal of Biometeorology. 2002;46(2):81–9. 12135203 10.1007/s00484-001-0119-6

[11] Bowman LR, Tejeda GS, Coelho GE, Sulaiman LH, Gill BS, McCall PJ, et al Alarm variables for Dengue outbreaks: A multi-centre study in Asia and Latin America. PLoS One. 2016;11(6):e0157971 doi: 10.1371/journal.pone.0157971 27348752 10.1371/journal.pone.0157971 PMC4922573

[12] Shewhart WA. Economic control of quality of manufactured product: ASQ Quality Press; 1931.

[13] Duclos A, Touzet S, Soardo P, Colin C, Peix J, Lifante J. Quality monitoring in thyroid surgery using the Shewhart control chart. British Journal of Surgery. 2009;96(2):171–4. doi: 10.1002/bjs.6418 19160350 10.1002/bjs.6418

[14] Rigau-Perez JG, Millard PS, Walker DR, Deseda CC, Casta-Vélez A. A deviation bar chart for detecting dengue outbreaks in Puerto Rico. American journal of public health. 1999;89(3):374–8. 10076488 10.2105/ajph.89.3.374 PMC1508601

[15] Organization WH. Operational guide: Early Warning and Response System (EWARS) for dengue outbreaks. 2017.

[16] Bowman LR, Runge-Ranzinger S, McCall P. Assessing the relationship between vector indices and dengue transmission: a systematic review of the evidence. PLoS Negl Trop Dis. 2014;8(5):e2848 doi: 10.1371/journal.pntd.0002848 24810901 10.1371/journal.pntd.0002848 PMC4014441

[17] Corbin J, Strauss A. Grounded theory research: Procedures, canons and evaluative criteria. Zeitschrift für Soziologie. 1990;19(6):418–27.

[18] Runge‐Ranzinger S, McCall P, Kroeger A, Horstick O. Dengue disease surveillance: an updated systematic literature review. Tropical Medicine & International Health. 2014;19(9):1116–60. 24889501 10.1111/tmi.12333 PMC4253126

[19] Wunderground. The Weather Channel, LLC. [Available from: http://www.wunderground.com.

[20] TuTiempo. Tutiempo Network, S.L. [Available from: http://en.tutiempo.net.

[21] El Centro Nacional de Programas Preventivos y Control de Enfermedades (CENAPRECE) M. Guía Metodológica para la Vigilancia Entomológica con Ovitrampas. CENAPRECE: MOH, Mexico; 2017.

[22] Campbell KM, Lin C, Iamsirithaworn S, Scott TW. The complex relationship between weather and dengue virus transmission in Thailand. The American journal of tropical medicine and hygiene. 2013;89(6):1066–80. doi: 10.4269/ajtmh.13-0321 23958906 10.4269/ajtmh.13-0321 PMC3854883

[23] Kroeger A MP, Ranzinger SR, Brady OJ, Olliaro P, Petzold M, et al. WHO-TDR Meeting. WHO-TDR. Liverpool. 2014.

[24] Runge-Ranzinger S, Kroeger A, Olliaro P, McCall PJ, Tejeda GS, Lloyd LS, et al Dengue contingency planning: from research to policy and practice. PLoS neglected tropical diseases. 2016;10(9):e0004916 doi: 10.1371/journal.pntd.0004916 27653786 10.1371/journal.pntd.0004916 PMC5031449

[25] Olliaro P, Fouque F, Kroeger A, Bowman L, Velayudhan R, Santelli A AC, et al Novel Tools and Strategies for the Prevention and Control of Arboviral Diseases: A Research-to-Policy Forum. PLOS NTD (in press); 2017. 10.1371/journal.pntd.0005967 PMC5794069 29389959

