# Supplementary material for: Early warning and response system (EWARS) for dengue outbreaks: Recent advancements towards widespread applications in critical settings
Source: PLoS One. 2018 May 4;13(5):e0196811. doi: 10.1371/journal.pone.0196811 (PMC5935393; doi:10.1371/journal.pone.0196811)
Supplement: S1 File — (PDF) [file pone.0196811.s001.pdf]

Evaluation of the prospective study on Early Warning and Response Systems.

1. Do you think that the alarm indicators used in the study are appropriate? Which others indicators do you think should be included?

2. Which alarm indicators were effective and which ones were not

3. Do you think the data collecting sheet is appropriate? What would you change?

4. Did you have problems filling in the data collecting sheet? Please describe.

5. Was there any issues/ problems in obtaining the data for the alarm indicators?

6. Do you think the graphs are easy to use and facilitate interpretation of the alert signals and outbreak detection? What would you change?

7. Do you think the response sheet (questionnaire) is appropriate? What would you change?

8. Was there adequate training provided prior to the study?

9. What do you think about the training course? Was the course appropriate? What would you change?

10. What do you think about the staged response (initial-early-late response)

11. Do you think the response activities defined for each stage are appropriate? What would you change?

12. Was there any issues/ problems in applying the control activities of the staged response?

13. What were the major difficulties when analyzing the data? Please describe.

14. Was the technical assistance appropriate to solve the issues?

15. Do you think the early detection and response tool help to reduce dengue outbreaks? What would you change?

16. What other benefits do you thing the early response and alert system can produce?

17. What are from your point of view the main weaknesses and strengths of the early warning and response tool?
